# Hemostasis using purse‐string reefing with endoscopic clip and endoloop for a huge hematoma after cold snare polypectomy

**DOI:** 10.1111/den.14263

**Published:** 2022-03-18

**Authors:** Ryoju Negishi, Takashi Muramoto, Ken Ohata

**Affiliations:** ^1^ Department of Gastrointestinal Endoscopy NTT Medical Center Tokyo Tokyo Japan

## Abstract

Watch a video of this article.

## Brief Explanation

Cold snare polypectomy (CSP) has become widely used as a safe technique for removing small colorectal polyps. Injury of submucosal arteries is suspected to be less frequent using CSP.^1^ Therefore, the rate of delayed bleeding tends to be much lower than that for hot snare polypectomy.^2^ We experienced a huge hematoma that formed after CSP; it was successfully managed using endoscopic purse‐string reefing.

**Figure 1:**
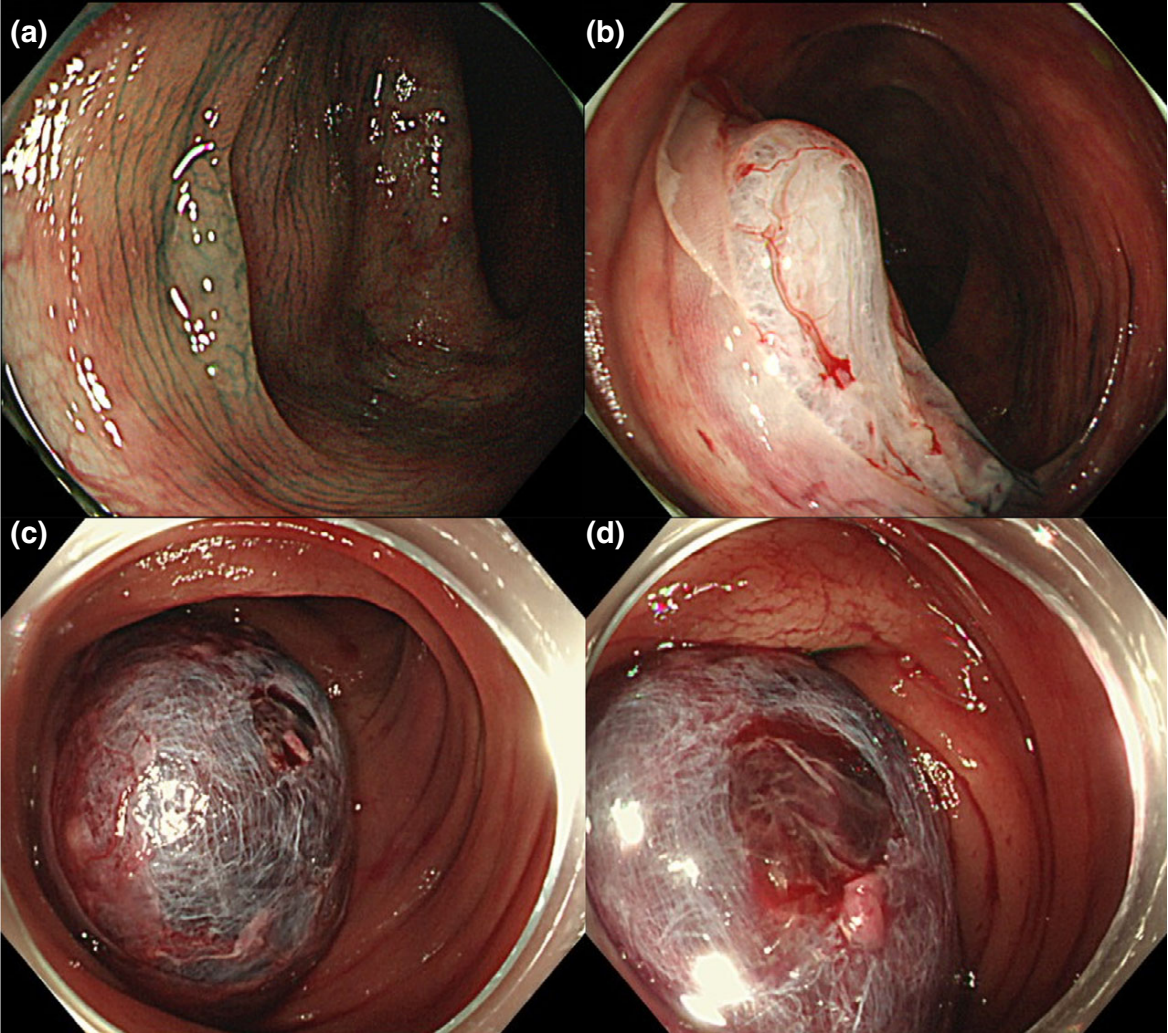
(a) Sessile serrated lesion, 8 mm in diameter, located at descending colon. (b) Post cold snare polypectomy site without bleeding and swelling by forcing water irrigation. (c) Huge hematoma which was formed at the post‐procedural ulcer floor. (d) The hematoma was covered by a thin submucosal tissue.

**Figure 2:**
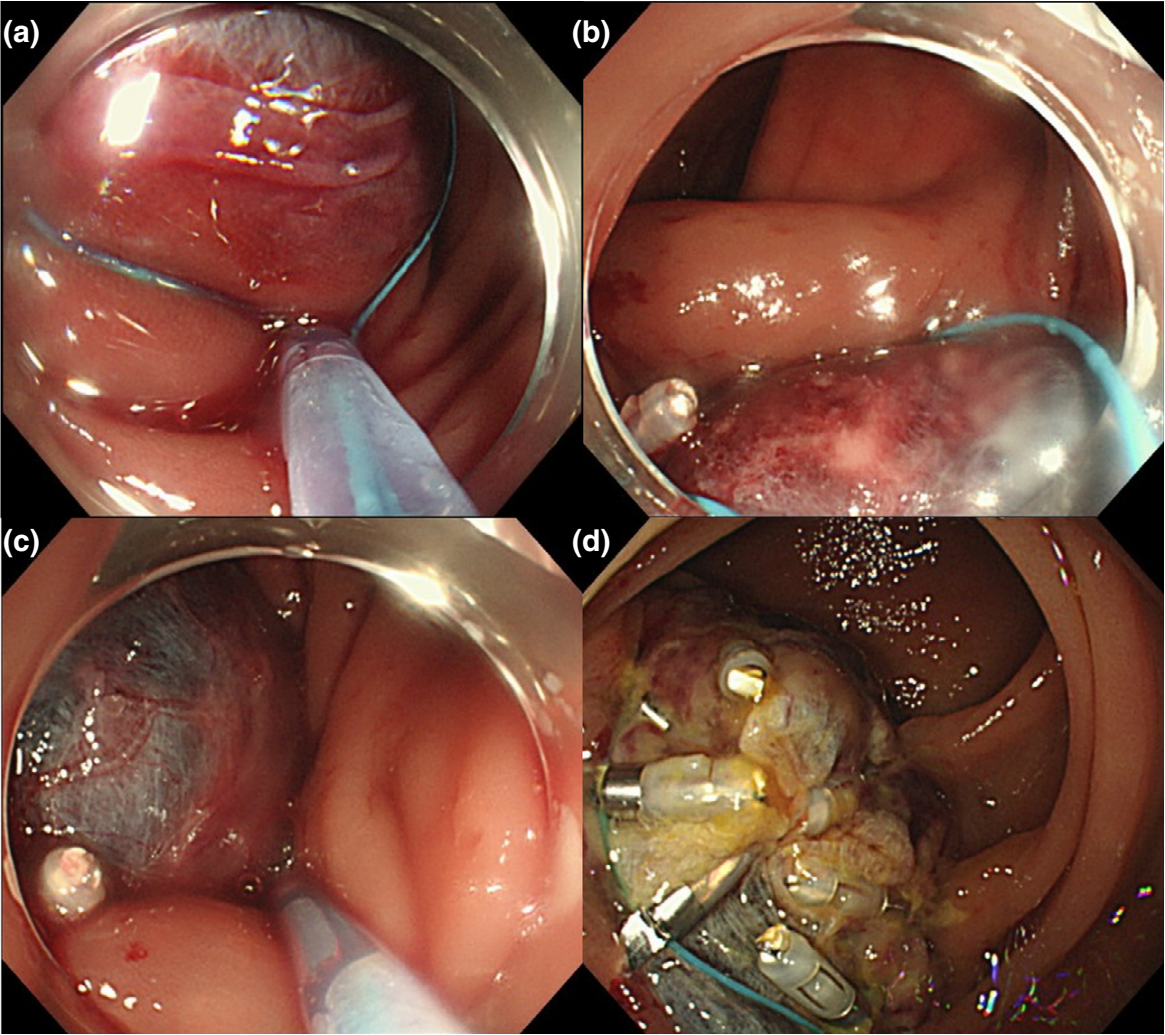
(a) An Endoloop was opened in line with the edge of the hematoma. (b) Fixed to the wall using clips. (c) Slowly tightened to strangulate the base of the hematoma. (d) Two days later, regression of the hematoma.

A 61‐year‐old woman underwent colonoscopy for screening. Patient history was unremarkable, no hemodialysis, normal platelet count, no antithrombotic medications, no coagulation abnormalities. A sessile serrated lesion (SSL), 8 mm in diameter, located in the descending colon was treated using CSP (Fig. 1a). Using a snare (SD‐400‐10; Olympus, Tokyo, Japan), the polyp was resected. Forced water irrigation over the exposed base was used to create a submucosal tamponade (Fig. 1b). On the following day, she had hematochezia. An urgent colonoscopy was conducted, and a huge hematoma covered by thin submucosal tissue was identified. Although a large volume of bleeding from the hematoma persisted (Fig. 1c,d), we could not identify the point of bleeding. Furthermore, the base of the hematoma was wide and the snare slipped, therefore the endoscopic purse‐string reefing method^3^ was applied. A detachable snare, 30 mm in diameter (Endoloop, HX‐400 U‐30; Olympus), was first opened in line with the edges of the hematoma and then fixed to the wall using clips (HX‐610‐090; Olympus) (Fig. 2a,b). Subsequently, Endoloop was slowly tightened to strangulate the base of the hematoma (Fig. 2c). Two days later, follow‐up endoscopy revealed the regression of the hematoma (Fig. 2d) (Video S1). There were no further complications after the procedure.

Because the resected layer during the CSP procedure is relatively shallow, an abundance of submucosal tissue tends to remain.^4^ In the present report, the remaining submucosal layer likely became the covering film and caused the formation of a huge nodular hematoma. However, there have been no reports of complications of huge hematoma after CSP.^5^ Although it is extremely rare, the possibility of severe bleeding after CSP should be taken into consideration, regardless of the low risk of delayed bleeding.

Authors declare no conflict of interest for this article.

## Supporting information


**Video S1** Successful endoscopic hemostasis using purse‐string reefing for a huge hematoma developing after a cold snare polypectomy.

## References

[1] Uraoka T , Ramberan H , Matsuda T , Fujii T , Yahagi N . Cold polypectomy techniques for diminutive polyps in the colorectum. Dig Endosc 2014; 26: 98–103. 24750157 10.1111/den.12252

[2] Horiuchi A , Ikuse T , Tnaka N *et al*. Cold snare polypectomy: Indication, devices, techniques, outcome and future. Dig Endosc 2019; 31: 372–7. 30549318 10.1111/den.13314

[3] Matsuda T , Fujii T , Emura F *et al*. Complete closure of a large defect after EMR of a lateral spreading colorectal tumor when using a two‐channel colonoscope. Gastrointest Endosc 2004; 60: 836–8. 15557972 10.1016/s0016-5107(04)02033-4

[4] Ito A , Suga T , Ota H *et al*. Resection depth and layer of cold snare polypectomy versus endoscopic mucosal resection. J Clin Gastroenterol 2018; 53: 1171–8. 10.1007/s00535-018-1446-2 29516270

[5] Takeuchi Y , Shichijo S , Uedo N *et al*. Safety and efficacy of cold versus hot snare polypectomy including colorectal polyps ≥1 cm in size. Dig Endosc 2022; 34: 274–83. 34324730 10.1111/den.14096

